# Effect of IRS4 Levels on PI 3-Kinase Signalling

**DOI:** 10.1371/journal.pone.0073327

**Published:** 2013-09-10

**Authors:** Gerta Hoxhaj, Kumara Dissanayake, Carol MacKintosh

**Affiliations:** 1 Medical Research Council Protein Phosphorylation Unit, College of Life Sciences, University of Dundee, Dundee, Scotland, United Kingdom; 2 Division of Cell and Developmental Biology, College of Life Sciences, University of Dundee, Dundee, Scotland, United Kingdom; Virgen Macarena University Hospital, School of Medicine, Spain

## Abstract

Insulin receptor substrate 1 (IRS1) and IRS2 are well-characterized adapter proteins that relay signals from receptor tyrosine kinases to downstream components of signalling pathways. In contrast, the function of IRS4 is not well understood. IRS4 overexpression has been associated with acute lymphoblastic leukaemia and subungual exostosis, while point mutations of IRS4 have been found in melanomas. Here, we show that while IRS4 expression is low in most cancer cell lines, IRS4 mRNA and protein levels are markedly elevated in certain cells including the NCI-H720, DMS114, HEK293T and HEK293AAV lines. Surprisingly, IRS4 expression was also strongly induced when HEK293 cells were infected with retroviral particles and selected under puromycin, making IRS4 expression a potential off-target effect of retroviral expression vectors. Cells with high expression of IRS4 displayed high phosphatidylinositol (3,4,5)-trisphosphate (PIP_3_) levels, as well as elevated Akt and p70 S6 kinase activities, even in the absence of growth factors. PI 3-kinase (PI3K) signalling in these cells depends on IRS4, even though these cells also express IRS1/2. Knockdown of IRS4 also inhibited cell proliferation in cells with high levels of IRS4. Together, these findings suggest IRS4 as a potential therapeutic target for cancers with high expression of this protein.

## Introduction

The insulin receptor substrate (IRS) proteins are a family of cytoplasmic adaptors that couple activation of the insulin receptor and other receptor tyrosine kinases to downstream PI3K–Akt and Ras signalling pathways [1], [2], [3], [4], [5]. Humans have IRS1, IRS2 and IRS4, while rodents also have IRS3, but the corresponding IRS3P in humans is a pseudogene. Based on the phenotypes of knockout mice, IRS1 and IRS2 have complementary roles in insulin and growth factor signalling, while IRS1 and IRS3 have complementary roles in adipogenesis [6], [7], [8]. In contrast, IRS4 is generally reported to be expressed at low levels, being picked up originally using sensitive phosphotyrosine antibodies in human embryonic kidney (HEK) cells and by PCR in rodent hypothalamus, where it functions in signalling from the insulin and leptin receptors [3], [9], [10], [11], [12]. IRS4 knockout mice exhibit mild defects in growth, reproduction and glucose homeostasis [13]. Overexpression of IRS4 rescues the effects of IRS1 and/or IRS2 knockout in rodent cells, and IRS4 levels were found to be increased during regeneration of resected rodent liver [14], [15], [16]. However, compared with rodents, the relative roles of the IRS proteins may be different in humans, which lack IRS3.

In humans, point mutations of IRS4 and overexpressions of IRS4 due to chromosomal translocations, were recently identified in human paediatric T-cell acute lymphoblastic leukaemia and subungual exostosis, a benign tumour of bone and cartilage in the distal phalanges of fingers and toes [17], [18], [19]. Somatic mutations of IRS4 were also found in melanoma cancer cells [20]. IRS4 has reported proliferative effects in human cell lines [21], [22]. IRS4 also interacts with adeno-associated viral proteins in infected cells and its expression is upregulated by adenoviral infection [23], [24].

In this study we show that while expression of IRS4 is generally low in the studied panel of cancer cell lines, it is high in NCI-H720, DMS114, HEK293T and HEK293AAV cells and that PI3K signalling in these cell lines relies on IRS4, but not IRS1. We also found that IRS4 expression is strongly induced upon infection of HEK293 cells with retroviral particles and subsequent selection with puromycin. Our findings also indicate that high expression of IRS4 has a significant role in PI3K signalling and therefore could be exploited to target this pathway in certain types of cancer.

## Results

### IRS4 expression in cancer cell lines

**Figure 1:**
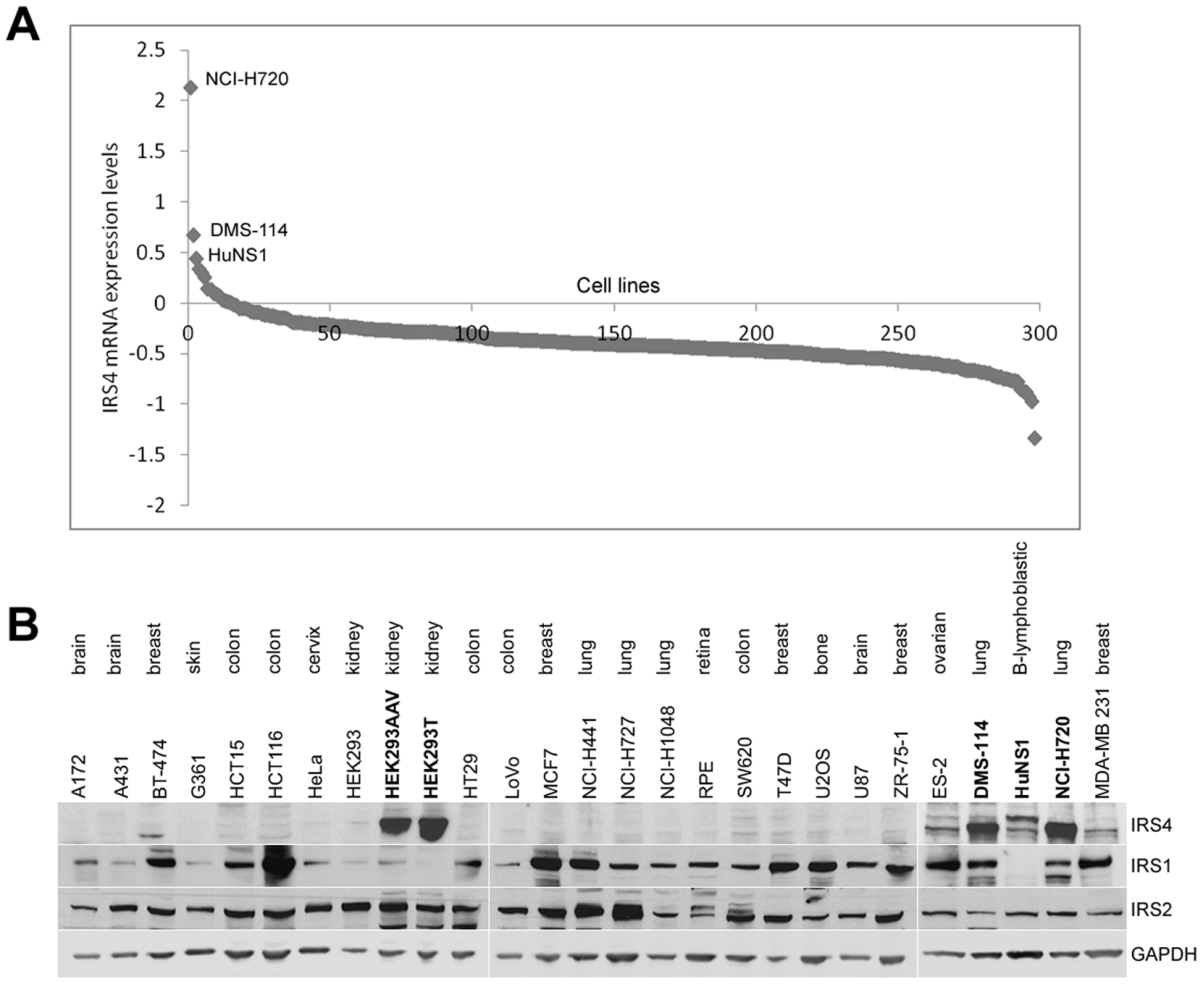
Expression of IRS4, IRS1 and IRS2 in various cell lines. (A) The graph represents the IRS4 mRNA expression in 298 cancer cell lines. Data in Oncomine (www.oncomine.com) was searched for mRNA expression in cell lines, normalised with multiple probe sets [38]. The higher the value, the higher the expression of IRS4 mRNA. (B) Cell lysates from the indicated cell lines were subjected to immunoblotting with the IRS4, IRS1, IRS2 and GAPDH antibodies

In comparison to other members of the IRS family, IRS4 is not as widely expressed [25]. However, IRS4 overexpression is associated with T-cell acute lymphoblastic leukaemia and subungual exostosis. We analyzed IRS4 mRNA expression in a panel of 298 cancer cell lines, in order to determine whether high levels of IRS4 expression is associated with certain types of cancers. We found that vast majority of cell lines analysed (283 out of 298) displayed low expression of IRS4 mRNA, if any at all (Fig. 1A, Table S1). However, 15 cell lines displayed moderate to high expression of IRS4 mRNA (Table S1). We then collected 27 cell lines, including four cancer cell lines with the highest mRNA expression levels, and checked for the expression of IRS1, IRS2 and IRS4 proteins by Western blotting. IRS4 protein was most highly expressed in four cell lines with high IRS4 mRNA levels, namely NCI-H720 (lung atypical carcinoid), DMS-114 (small cell lung carcinoma), HEK293AAV (HEK293 cells that contain adeno-associated virus) and HEK293T (HEK293 cells harbouring the SV40 virus T-antigen), though not the parental HEK293 cells (Fig. 1B). Lower, but detectable, levels of IRS4 were also seen in HuNS1 (multiple myeloma) and ES-2 (ovarian clear cell adenocarcinoma) cells (Fig. 1B). Compared with IRS4, IRS1 and IRS2 were more ubiquitously expressed in the cell lines tested (Fig. 1B). Elevated IRS4 expression was not associated with any particular cancer type, since high expression of IRS4 mRNA was found only in a small set of cell lines that had been derived from different cancer types.

### PI3K signalling in cells with high IRS4 expression employs IRS4, but not IRS1

**Figure 2:**
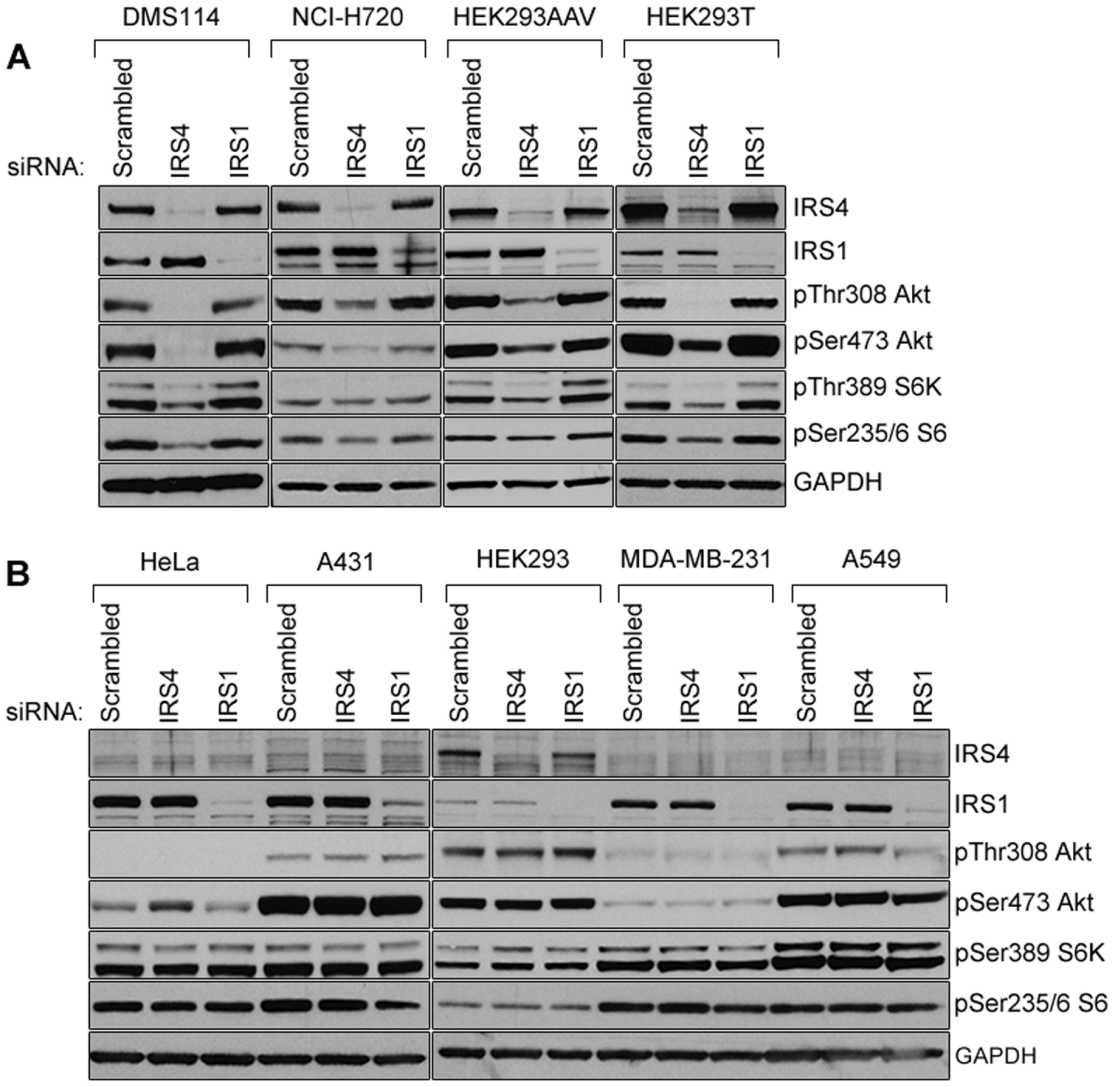
Effects of IRS1 and IRS4 knockdown on the PI3K pathway in cells with low and high IRS4 protein levels. (A) Cell lines exhibiting high levels of IRS4 such as DMS114, NCI-H720, HEK293AAV, HEK293T were transfected with 100 nM final concentration of control (Scrambled), IRS4 or IRS1 siRNAs using Dharmafect 1 transfection reagent following manufacturer's instructions. After 48 h, cells were harvested and lysates (20 µg) were immunoblotted using the indicated antibodies to test the activation status of the PI3K pathway. (B) As in (A) except that the experiment was performed in cells with low expression of IRS4 such as HeLa, A431, HEK293, MDA-MB-231 and A549.

**Figure 3:**
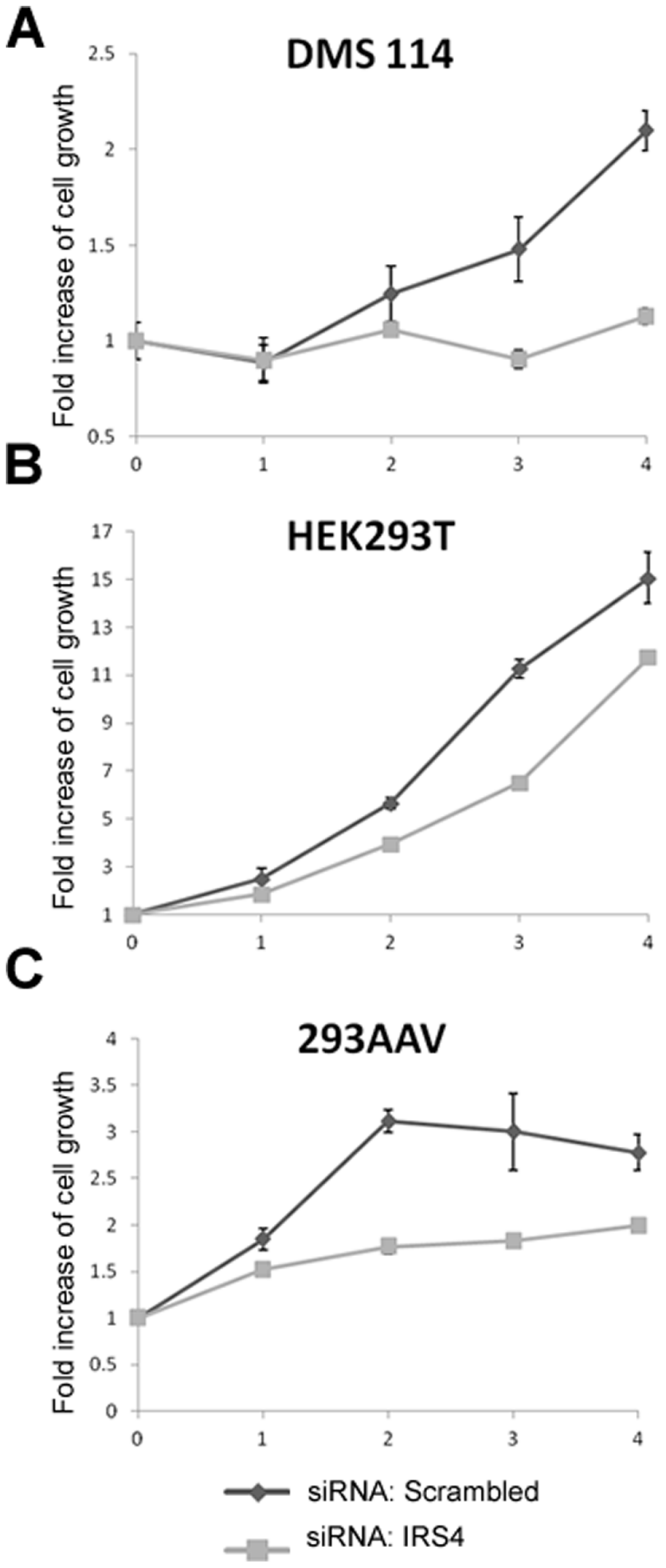
Effect of IRS4 knockdown on growth of DMS114, HEK293T and 293AAV cells. (A) DMS114, (B) HEK293T and (C) 293AAV cells were transfected with control (Scrambled) and IRS4 siRNA oligos. At 24 h post transfection, cells were seeded in 24-well plates (20 000 cells per well). For each time point, cells were washed with phosphate-buffered saline (PBS), fixed in 4% (v/v) paraformaldehyde in PBS for 15 min, washed in water once, stained with 0.1% Crystal Violet in 10% ethanol for 20 min and washed thrice with water. Crystal Violet was extracted from cells with 0.5 ml of 10% (v/v) acetic acid for 20 min while shaking at room temperature and the absorbance measured at 590 nm.

To establish whether the high IRS4 expression in the aforementioned four cell lines is functional, we performed siRNA-mediated knockdown of IRS4 or IRS1 (Fig. 2A). We used five commonly-used cell lines with low or no IRS4 expression as negative controls (HeLa, A431, HEK293, MDA-MB-231 and A549) (Fig. 2B). In DMS114, NCI-H720, HEK293AAV and HEK293T cells, the knockdown of IRS4, but not IRS1, caused a marked decrease in phosphorylation of Ser473 and Thr308 of Akt, as well as Thr389 of p70 S6 kinase (S6K) and Ser235/6 of the S6K substrate S6, suggesting a general decrease in activation of the PI3K pathway. We concluded that in these cell lines IRS4, but not IRS1, is employed to mediate PI3K signalling, even though both proteins are expressed (Fig. 2A). In contrast, knocking down IRS4 (assuming low expression of IRS4 was present) or IRS1 in HeLa, A431, HEK293, MDA-MB-231 and A549 cells did not affect activation of the PI3K pathway (Fig. 2B). We also found that depleting IRS4 in DMS114, HEK293T and HEK293AAV cells decreased cell proliferation (Fig. 3). NCI-H720 cells grow as a suspension of aggregated cells, which precludes reliable quantitation of their cell proliferation rate.

### HEK293 clones with increased IRS4 expression have elevated PI3K signalling

**Figure 4:**
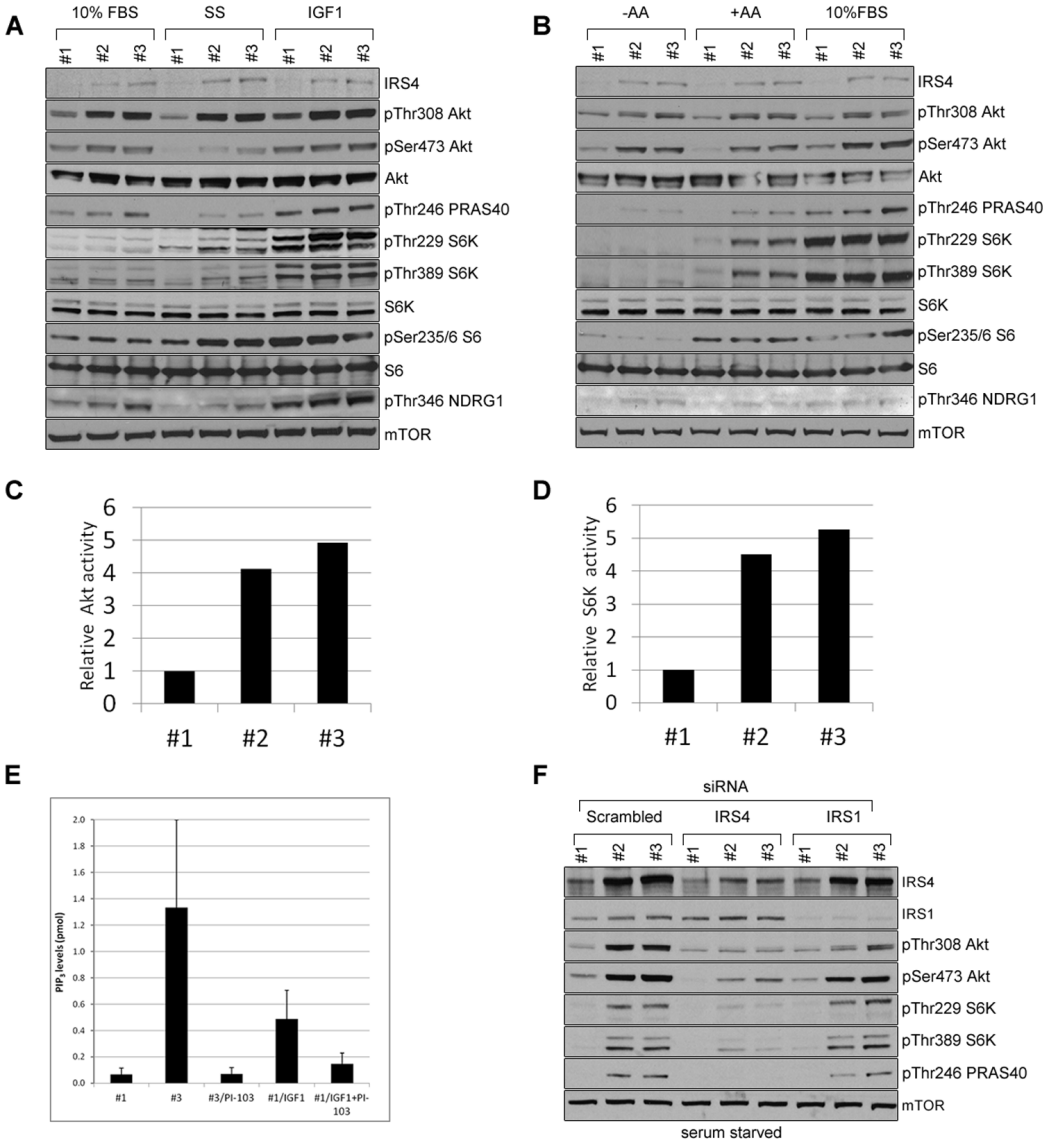
HEK293 clones with increased IRS4 expression have elevated PI3K signalling. (A) Clones of HEK293 cells expressing different levels of IRS4 (#1-low, #2- and #3-high) obtained after retroviral infection and puromycin selection as described in materials and methods, were serum starved and then stimulated with IGF1 or 10% FBS. Cell lysates (30 µg) were subject to immunoblotting with the indicated antibodies. (B) As in (A), except that cells were starved of amino acids and then stimulated with amino acids or 10% FBS for 20 min. (C and D) The cells expressing different levels of IRS4 were serum deprived overnight and assayed for the catalytic activity of immunoprecipitated endogenous Akt and S6K1 using Crosstide peptide as substrate, as described in materials and methods. (E) Clones #1 and #3 of HEK293 cells were serum starved overnight and assayed for PIP_3_ as in [37]. Cells from clone #1 were serum starved and treated with IGF1+/− PI-103 (1 µM, 30 min) and used as positive and negative controls, respectively. (F) Cells expressing low (#1) or high (#2 and #3) levels of IRS4 were transfected with control, IRS4 or IRS1 siRNA oligos and the activity of PI3K pathway components was analysed as in (A) under serum starved conditions.

HEK293 cells have very low IRS4 protein levels (Fig. 1B). We noticed however, that IRS4 protein levels were increased in some HEK293 clones that had been infected with retroviral particles and selected with puromycin. To further explore this correlation, such cells were selected and graded as #1 for lower IRS4 expression, and #2 and #3 for higher IRS4 expression. A higher IRS4 level was accompanied by an increase in phosphorylation and activity of Akt and S6K (Fig. 4 A–D). Even under serum deprivation, HEK293 clones with high levels of IRS4 displayed elevated phosphorylation of Ser473 and Thr308 of Akt, Thr246 of PRAS40 (Akt substrate), Thr346 of NDRG1 (substrate of serum/glucocorticoid regulated kinase 1 (SGK1)), Thr389 and Thr229 of S6K, and Ser235/6 of S6 (Fig. 4 A and B). Also, even when starved of amino acids, HEK293 clones with high IRS4 levels still displayed high phosphorylation of Ser473 and Thr308 of Akt, and Thr246 of PRAS40, though not of S6K (Fig. 4B). However, amino acid addition resulted in higher phosphorylation of S6K in HEK293 clones with high expression of IRS4 compared to HEK293 clones with low IRS4 levels (Fig. 4D). The kinase activities of Akt and S6K were 4 to 5 times higher in HEK293 clones with high IRS4 levels, compared to HEK293 clones with low IRS4 levels, even under conditions of serum deprivation (Fig. 4 C and D). PIP_3_ levels were also markedly elevated in HEK293 clones with high IRS4 levels. Notably, PIP_3_ levels in these cells are more than 3-fold higher than in normal HEK293 cells stimulated with IGF1 (Fig. 4E). The elevated PIP_3_ levels in HEK293 clones with high IRS4 levels were suppressed by the PI3K inhibitor PI-103 (Fig. 4E).

To test whether the increases in phosphatidylinositol (3,4,5)-trisphosphate (PIP_3_) levels, and Akt and S6K activities, were due to IRS4, we knocked down IRS4 or IRS1 using siRNA. Figure 4F and Fig. S1 shows that depletion of IRS4, but not IRS1, decreased PI3K signalling, suggesting that the elevated signalling in the HEK293 clones with high IRS4 levels was specific to IRS4 and not IRS1.

### Overexpression of IRS4 in U2OS cells activates PI3K signalling

**Figure 5:**
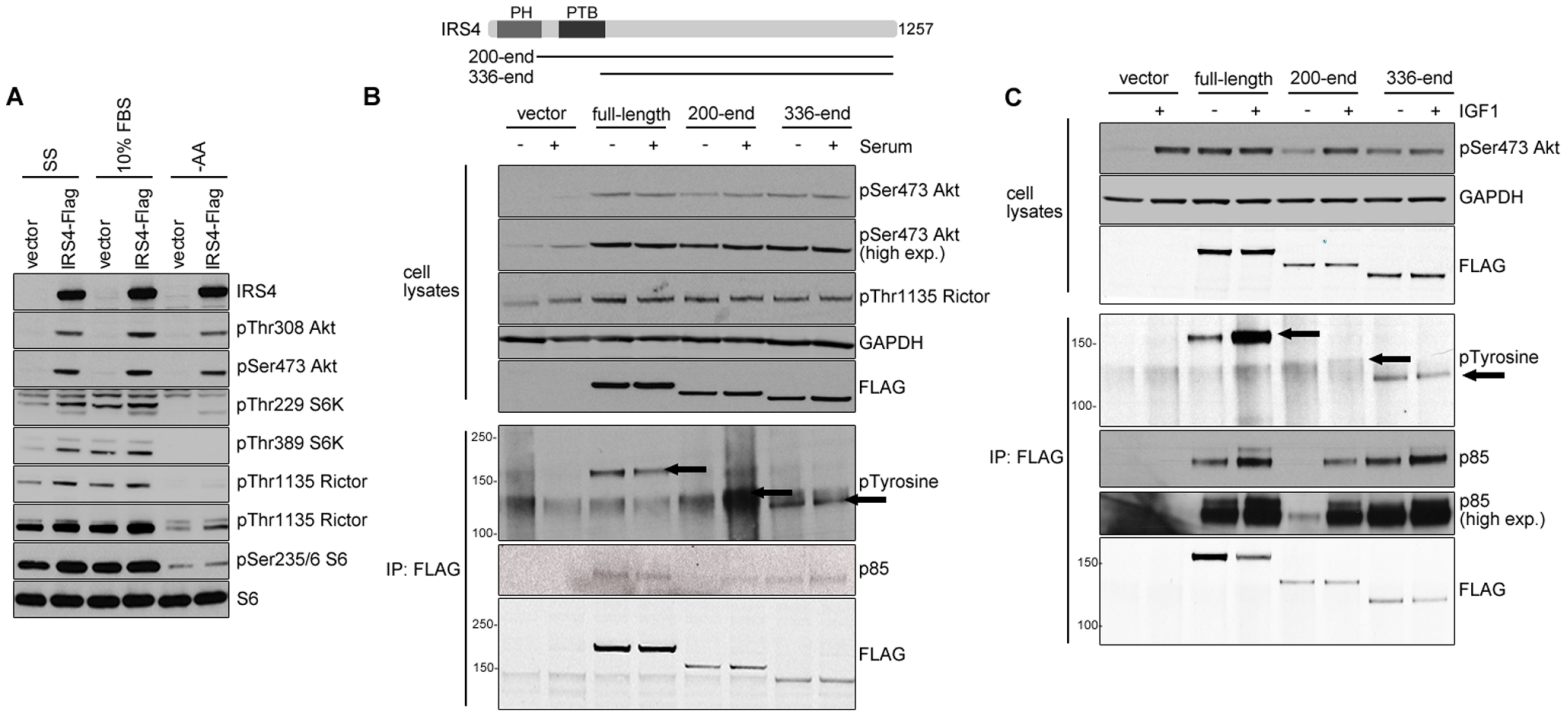
The effects of IRS4 overexpression on the PI3K pathway. (A) U2OS-Flpin™ cells expressing empty vector or IRS4-FLAG protein were analysed for the activation of PI3K pathway components under serum starved (SS), amino acid-starved (-AA) and 10% FBS conditions. Cells were deprived of serum for 12 h (SS) and starved of amino in EBSS for 2 h (-AA). (B) U2OS-Flpin™ cells expressing empty vector, full length IRS4-FLAG (WT), and the indicated fragments containing residues from 200–end-IRS4-FLAG or 336–end-IRS4-FLAG proteins were analysed for the activation of PI3K pathway components, in the presence or absence of serum. (C) As in B, except that the cells were treated with IGF1 (50 ng/ml, 20 min). The FLAG tagged proteins expressed were analysed for the Tyrosine phosphorylation as well as the binding to the p85 regulatory subunit of PI3 kinase.

U2OS-FlpIn™ cells stably expressing FLAG-tagged IRS4 also displayed strongly enhanced PI3K signalling, compared to control empty vector cells (Fig. 5A). Again, the phosphorylation of Akt in these cells was not decreased by serum- or amino acid-deprivation, whereas phosphorylation of S6K was decreased under amino acid starvation (Fig. 5A). Interestingly, this effect of IRS4 overexpression was not dependent on its PH (pleckstrin homology) or PTB (phosphotyrosine binding) domains (Fig. 5B and 5C). Expressing fragments of IRS4 that lack the PH domain (residues 200-end) or the IRS-type PTB domain (residues 336-end) also caused enhanced phosphorylation of Ser473 Akt and pThr1135 Rictor (S6K substrate) (Fig. 5B and 5C). We found that full-length IRS4 was tyrosine phosphorylated even under serum-starved conditions and this was further increased in response to IGF1, but not 10% serum (Fig. 5B and 5C). Moreover, IRS4 bound to p85 subunit of the PI3K under serum-starved conditions (Fig. 5B, lane 3 and Fig. 5C, lane 3). We found that in HEK293T cells, which have high IRS4 levels, endogenous IRS4 and IRS1 are tyrosine-phosphorylated in response to IGF1, but not serum (Fig. S2). These data suggest that high levels of IRS4 are sufficient to activate the PI3K pathway to the levels of activation mediated by stimulation of cells with IGF1 (50 ng/ml for 20 min), which provides key evidence that high IRS4 expression is important for the activation of this pathway in these cells.

**Figure 6:**
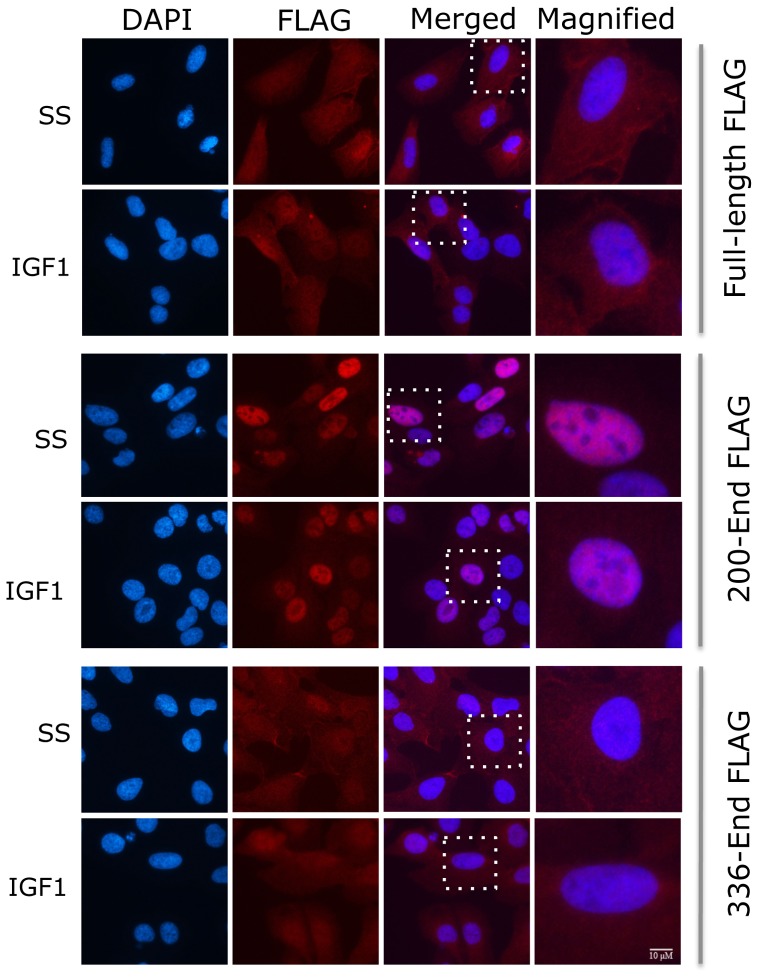
Deletion of PH domain S4 targets IRS4 to the nucleus. U2OS-Flpin™ cells expressing full length IRS4-FLAG and the indicated fragments containing residues from 200–end-IRS4-FLAG or 336–end-IRS4-FLAG proteins were analysed for their subcellular localization, under serum starved (SS) or addition of IGF1(50 ng/ml, 20 min). Cells were stained for FLAG antibody and with DAPI as described in Materials and Methods.

Interestingly, IRS4 mutants lacking PH, or PH and PTB domains showed some tyrosine phosphorylation and also interacted with the p85 subunit of PI3K, and these interactions were enhanced by IGF1, although no increase in tyrosine phosphorylation was observed in response to IGF1 or 10% serum. The tyrosine phosphorylation of the mutant lacking PH domain was more difficult to assess since the signal interfered with an unspecific band recognized by the phosphotyrosine antibody. Strikingly, full length IRS4 or IRS4 lacking both PH and PTB domain were localized to the cytoplasm and plasma membranes under both serum starved and IGF1-stimulated conditions, whereas IRS4 lacking only the PH domain was localized to the nucleus under serum starved conditions (Fig. 6). However, the PH deletion mutant was also visualised in the cytoplasm, consistent with its increased binding to the p85 PI3K subunit in response to IGF1 (Fig. 6 and Fig. 5C).

### The 2R-WGD and evolution of the IRS protein family in the vertebrate animals

**Figure 7:**
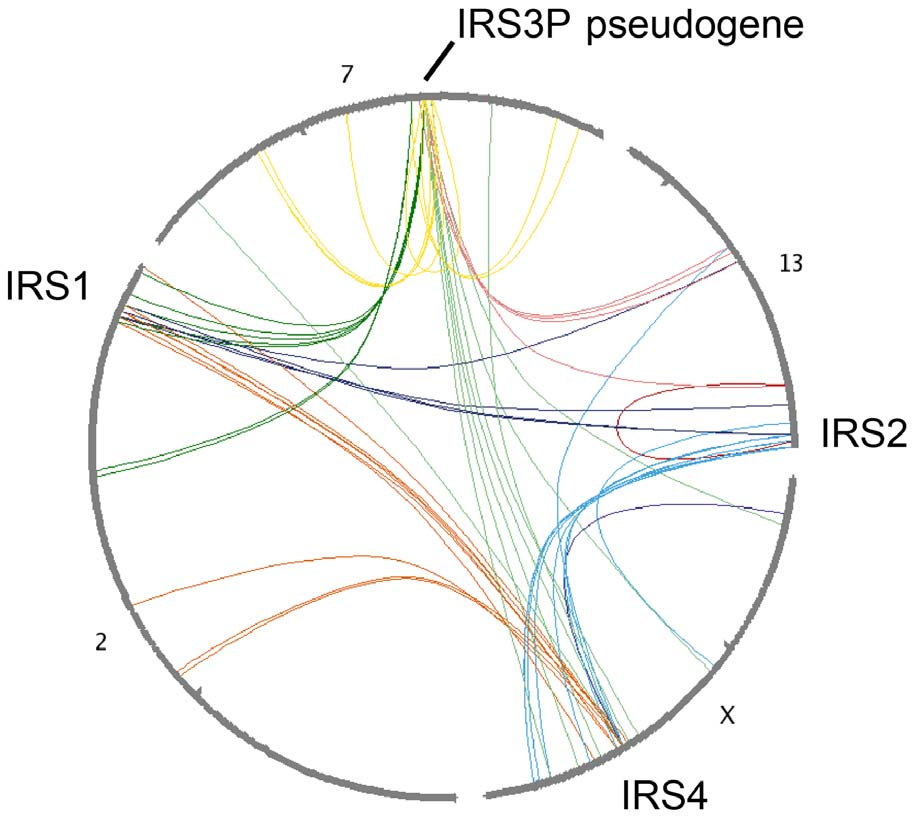
Gene synteny analyses of human IRS1, IRS2, IRS4 and the IRS3P pseudogene. A circle plot links the human gene synteny clusters containing human IRS1, IRS2, IRS4 genes and the IRS3P pseudogene. Plots were generated using the Synteny Database (teleost.cs.uoregon.edu/synteny_db) [39] with the *Branchiostoma* genome as outgroup. Arcs around the circumference represent chromosomes, while those within the circle connect pairs of related genes. The human IRS1 (chromosome 2q36), IRS2 (13q34) and IRS4 (Xq22.3) genes and also genes within 500 kb of these three genes, were used as queries. Different chromosomes were tested in the fourth position until a strong synteny trace was observed for chromosome 7 as shown, and these synteny lines were then confirmed to converge on the human IRS3P pseudogene at 7q22.1.

Considering that the relative roles of IRS4 may differ in the rodents used for genetic studies, and humans, which lack IRS3, we wished to define how this protein family evolved. The vertebrate IRS proteins displays the genetic signature of a family of 2R-ohnologues, which are protein families generated by the two rounds of whole-genome duplication (2R-WGD) at the origin of the vertebrates [26], [27], [28]. Thus, the four human genes are on different chromosomes, with strong pairwise synteny between genes on chromosome 2q36 (the location of the IRS1 gene) and 7q22.1 (IRS3P pseudogene), and between the 13q34 (IRS2) and Xq22.3 (IRS4) regions (Fig. 7). Also, a single IRS (C3ZU02_BRAFL) was identified in *Branchiostoma floridae*, the basal-most chordate invertebrate related to the vertebrates. A plausible interpretation is that a single invertebrate pro-orthologue gave rise to two genes in the first round of WGD during early vertebrate evolution: One of these genes generated IRS1 and IRS3 in the second WGD, with later conversion of IRS3 to a pseudogene in primates, but not rodents. Duplication of the other gene generated IRS2 and IRS4. Unfortunately, we failed to find sufficient IRS sequence data to deduce when psuedogenization of IRS3 originated in the primate lineage. Pinpointing when IRS3 protein was lost should give clues about how this event affected the relative roles of IRS1, 2 and 4.

A recent evolutionary analysis placed IRS5/Dok4 (downstream of kinase-4) and IRS6/Dok5 in the same family as IRS1, IRS2, IRS3 and IRS4 [29]. However, while the chromosomal regions containing Dok4 (16q21), Dok5 (20q13.2) and Dok6 (18q22.2) share synteny with each other (not shown), they share no obvious synteny with the regions encoding IRS1, 2, 3 and 4. Thus, IRS1/2/3/4 and Dok4/5/6 are two separate 2R-ohnologue families.

## Discussion

Our results caution that IRS4 expression is a potential off-target effect of retroviral delivery of genetic material into cells, and that high IRS4 levels identified in certain cancer cells can drive signalling via PI3K even in the absence of growth factors. Our findings suggest that cells with high levels of IRS4 use this protein for activation of PI3K signalling, even though they also contain IRS1 and IRS2. Perhaps, cells expressing IRS4 build an accompanying regulatory network that cannot accommodate IRS1 and/or IRS2, so that this network collapses when the IRS4 is subsequently removed. An alternative suggestion is that the expression of IRS4 triggers a feedback mechanism that inhibits the action of IRS1 and IRS2 [30], and that this putative inhibitory mechanism does not disappear when IRS4 is removed. One way to distinguish between these possibilities might be to identify regulatory proteins that interact with each IRS protein. In addition to viral proteins, IRS4 interacts with the WD repeat protein WDR6 in rat brain; the non-receptor tyrosine kinase breast tumor kinase (Brk) in HEK293 cells; protein phosphatase 4, which is proposed to be involved in downregulating IRS4 in response to tumour necrosis factor-alpha; and ankyrin repeat and SOCS box containing protein 4 (Asb-4) [12], [31], [32], [33]. However, the relevance of these for the effects of IRS4 observed here is unclear.

We found that when IRS4 is overexpressed in cells, the PI3K pathway is activated to the extent seen upon stimulation of cells with IGF1 (50 ng/ml for 20 min) (Fig. 5C). Strikingly, IRS4-mediated activation was not dependent on growth factors, since it was seen even in serum-starved cells (Fig. 5B and 5C). Moreover, IRS4 was tyrosine-phosphorylated and interacted with the p85 subunit of PI3K even under serum-starved conditions (Fig. 5B and 5C), suggesting that basal receptor tyrosine kinase (RTK) activation is sufficient for IRS4-mediated activation of the PI3K pathway under these conditions. Interestingly, IRS4 mutants lacking PH, or PH and PTB domains, also interacted with the p85 subunit of the PI3K, and these interactions were enhanced by IGF1 (Fig. 5C). However, we observed no IGF1-stimulated increase in tyrosine phosphorylation of the IRS4 mutant lacking the PTB domain, consistent with binding of IRS4 to the RTK being critical for its growth factor-stimulated tyrosine phosphorylation (Fig. 5C, last two lanes).

Lack of the PH domain (up to residue 200) caused IRS4 to be localized predominantly in the nucleus (Fig. 6) with a lesser activation of PI3K in the absence of growth factor relative to that seen with full-length IRS4. However, when both the PTB and PH domains were missing, cytoplasmic location and activation of PI3K signaling in the absence of growth factors were restored to the levels seen with the overexpressed full-length IRS4 (Fig. 5B and 5C). IRS4 has a putative nuclear localization sequence (NLS) at residues 199 and 208 between the PH and PTB domains (http://nls-mapper.iab.keio.ac.jp/cgi-bin/NLS_Mapper_form.cgi). We therefore suggest that the PH domain is inhibitory to the NLS, explaining why IRS4 lacking the PH domain is localized to the nucleus. On the other hand, deletion of PTB domain as well as PH domain results in loss of the NLS sequence; hence this IRS4 fragment is localized to the cytoplasm. Notably, a mutant form of IRS4 (deletion from aa24–aa221, lacking the PH domain and putative NLS) was identified in a case of paediatric T-cell acute lymphoblastic leukaemia [17]. The authors were unsure of the implications of their findings, expecting that such a deletion might decrease PI3K signalling. However, our data suggest that the mutant protein in that leukaemia patient may be active.

Previously, IRS4 levels were shown to be elevated after adenovirus 5 E1A infection and transfections of cells [24]. Indeed, in searching for cell lines with high IRS4 expression, we noticed that the levels of IRS4 were markedly higher in HEK293 cells stably expressing the T antigen (293T) and adeno-associated virus (293AAV) than in parental HEK293 cells. We also found that IRS4 levels could increase as a result of retrovirus infection and selection by puromycin in HEK293 cells. This makes IRS4 expression a potential ‘off target’ effect of the commonly-used retroviral delivery of DNA into cells. The retroviral genome encodes the Gag, Pol and Envelope proteins. In contrast to the effects of retrovirus infection, expressing only the Gag and Pol proteins had no obvious effect on cellular IRS4 expression (data not shown). Neither was IRS4 expression increased in cells transfected to express the AAV viral coat protein, nor in HuH7 cells transfected with hepatitis C RNAs (data not shown). Perhaps, a prolonged expression of these viral proteins (instead of transient transfection) is required to promote high levels of IRS4. The finding that retrovirus infection followed by puromycin selection is an important factor in elevating the IRS4 levels (data not shown), suggests that IRS4 protein levels might be required for selecting for clones with higher survival rates under stress conditions. Other studies have shown that viral oncoproteins associate with and use IRS proteins to turn on pro-survival signals during virus transformation. For example, one mechanism by which the SV40 T-antigen transforms cells is by associating with IRS1 [34], and inactivation of IRS1 causes a delay in the transformation of the SV40 T-antigen [35].

## Materials and Methods

### Materials

Protease inhibitor cocktail tablets (#1697498) were from Roche; PEI from Polysciences; Dharmafect 1 from Thermo Scientific; Protein G-Sepharose and enhanced chemiluminescence Western blotting kit were from Amersham Bioscience; insulin, transferrin, sodium selenite, hydrocortisone, beta-oestradiol and amino-6-methylmercaptopurine were from Sigma. Precast NuPAGE polyacrylamide Bis-Tris gels, Colloidal Coomassie, LDS sample buffer, hygromycin, tetracycline, zeocin, GIBCO® Earle's Balanced Salt Solution (EBSS) and Gibco® MEM Amino Acids Solution 50 were from Invitrogen; sequencing-grade trypsin was from Promega; and microcystin-LR was kindly provided by Dr. Linda Lawton (Robert Gordon University, Aberdeen, UK).

### Antibodies

The IRS4 antibody was from Santa Cruz Biotechnology (sc-28830) and IRS2 antibody from Millipore (MABS15). Antibodies that recognize pSer473 Akt, pThr308 Akt, pThr389-S6K, pSer235/6-S6, pThr1135-rictor, phosphotyrosine, GAPDH and IRS1 were from Cell Signaling Technology. Anti-FLAG M2 was from Sigma. Anti-Akt was raised in sheep (S695B) against the RPHFPQFSYSASGTA peptide (residues 466–480 of human Akt); and anti-S6K was raised in sheep (S417B) against the peptide AGVFDIDLDQPEDAGSEDEL (residues 25–44 of human S6K). Antibody production and purification were arranged via the Division of Signal Transduction Therapy (DSTT) of the University of Dundee.

### Stimulation of cells with amino acids

Cells were starved of amino in EBSS for 2 h (-AA). A mixture of amino acids (50X Gibco® MEM Amino Acids Solution) was used at 1× concentration for 10 min.

### Cell lysis

Cells were lysed in 0.35 ml per 10 cm dish in ice-cold Triton X-100 Lysis Buffer comprising 25 mM Tris-HCl (pH 7.5), 1 mM EDTA, 1 mM EGTA, 1% Triton X-100, 50 mM NaF, 5 mM sodium pyrophosphate, 1 mM sodium orthovanadate, 1 mM benzamidine, 0.2 mM PMSF, 0.1% 2-mercaptoethanol, 1 μM microcystin-LR, 0.27 M sucrose and one mini Complete™ protease inhibitor cocktail tablet (#1697498, Roche) per 10 ml of lysis buffer. Lysates were clarified by centrifugation (16,000× g at 4°C for 15 min); snap frozen and stored at −80°C. Protein concentrations were determined with Coomassie Protein Assay Reagent (Thermo Scientific).

### Cell proliferation assay

Cells were transfected with control (Scrambled) and IRS4 siRNA oligos. At 24 h post-transfection, cells were seeded in 24-well plates (20000 cells per well). For each time point, cells were washed with PBS, fixed in 4% (v/v) paraformaldehyde in PBS for 15 min, washed with water once, stained with 0.1% Crystal Violet in 10% ethanol for 20 min and washed thrice with water. Crystal Violet was extracted from cells with 0.5 ml of 10% (v/v) acetic acid for 20 min while shaking at room temperature and the absorbance measured at 590 nm.

### Plasmids

Recombinant DNA procedures, restriction digests, ligations and PCR were performed using standard protocols by Dr Rachel Toth in the DSTT. All PCR reactions were carried out using KOD Hot Start DNA polymerase (Novagen). DNA sequencing was performed by The Sequencing Service, College of Life Sciences, University of Dundee (www.dnaseq.co.uk). Vectors for expression of IRS4 forms were pcDNA5 FRT/TO modified to express N-terminally FLAG-tagged forms of human IRS4 proteins. In-house numbers for the reference clones are as follows: FLAG-IRS4 (DU36186), FLAG-(aa 200-R1257)-IRS4 (DU36209) and FLAG-(aa 336-R1257)-IRS4 (DU 36210). The vectors containing shRNA Scrambled were pSuper.retro.puro (DU30730).

### Generation of stably-transfected U2OS Flp-In stable lines

U2OS-Flpin™ cells maintained in DMEM containing 10% FBS and 2 mM L-glutamine, were transfected with pcDNA5-FRT-TO vectors to express IRS4-FLAG or IRS4-FLAG fragments together with the plasmid pOG44 (Invitrogen), which encodes Flp recombinase. The pOG44 to pcDNA5-FRT-TO vector ratio was (9∶1). At 48 h post transfection, the cells were selected in DMEM growth medium containing 100 μg/ml hygromycin B.

### Kinase activity assays

Kinase assays were performed as in [36]. Briefly, endogenous Akt and S6K were immunoprecipitated from 0.25 mg of cell lysate. The lysates were incubated with 1 µg of Akt or S6K antibody and 15 µl of protein G-Sepharose beads for 2 h at 4°C on a shaking platform. The immunoprecipitates were washed twice with lysis buffer containing 0.5 M NaCl, followed by two washes with kinase buffer containing Tris-HCl (pH 7.5), 0.1 mM EGTA and 0.1% 2-mercaptoethanol. Kinase reactions (40 µl) contained 0.1 mM [γ-^32^P] ATP (∼230 cpm/pmol), 5 mM magnesium acetate, 0.1% 2-mercaptoethanol and 30 mM Crosstide peptide (GRPRTSSFAEGKK). Kinase reactions were performed at 30°C for 20 min on a shaking platform, and were stopped by spotting on to P81 phosphocellulose paper, which was washed thrice in phosphoric acid, rinsed in acetone and air-dried before Cerenkov counting.

### PIP_3_ assay

Phosphatidylinositol (3,4,5)-trisphosphate (PIP_3_) measurements were kindly performed by Dr. Alexander Gray (University of Dundee) as previously described [37].

### Immunofluorescence of fixed cells

Cells grown on coverslips were fixed with 4% paraformaldehyde, permeabilized with 0.2% Triton X-100 for 5 min, rinsed with PBS, blocked in 10% donkey serum in PBS for 30 min and incubated with primary antibody (FLAG-M2 (1 µg/ml)) in 1% BSA/PBS overnight at 4°C. The coverslips were washed 3 times with 1% BSA/PBS and incubated with secondary antibodies (Alexa Fluor® 594 Donkey Anti-Mouse IgG (1∶500)) for a further hour at room temperature in the dark. After 3 washes with 1% BSA in PBS, cells were stained with DAPI (4′, 6-diamidino-2-phenylindole) and mounted using Vectashield (Vector Laboratories). Cells were visualized with a Nikon Eclipse Ti-S fluorescence microscope at 40× magnification. Images were captured by NIS-Elements software and processed using Adobe Photoshop.

### Maintenance of cell lines

All cells were maintained at 37°C in a humidified atmosphere with 5% CO_2_. NCI-H720, HuNS1, DMS114 and ES-2 cells were obtained from ATCC. NCI-H720 cells were maintained in DMEM: F12 medium containing 5 µg/ml insulin, 10 µg/ml transferrin, 30 nM sodium selenite, 10 nM hydrocortisone, 10 nM beta-oestradiol, 2 mM L-glutamine and 5% FBS; HuNS1 cells were in RPMI 1640 medium containing 2 mM L-glutamine, 4.5 g/l glucose, 10 mM HEPES, 1 mM sodium pyruvate, 1.5 g/l sodium bicarbonate, 20 µM 2-amino-6-methylmercaptopurine and 15% FBS; DMS114 were maintained in Waymouth's MB medium containing 10% FBS; ES-2 cells in minimum essential medium Eagle alpha modification (αMEM) supplemented with 5% FBS; and HEK293, HEK293AAV, HEK293T, HeLa and U2OS cells were grown in DMEM containing 10% FBS supplemented with 2 mM L-glutamine and 1% penicillin/streptomycin.

### Retroviral infection

H29, a packaging cell line for retrovirus production, was maintained in DMEM containing 10% FBS, 2 μg/ml puromycin, 0.3 mg/ml G418 and 0.5 µg/ml tetracycline. Before transfection, the cell medium was changed to DMEM containing 10% FBS only. Cells grown in T75 flasks to 50% confluency were transfected with 15 µg of shRNA retroviral vector (pSuper-retro.puro) and 45 μl PEI in 2 ml Opti-MEM. After 48 h, viruses were collected, filtered (0.45 μm pore size) and used to infect HEK293 cells in the presence of 5 μg/ml polybrene. At 24 h post infection, the culture medium was replaced and cells were selected in DMEM containing 10% FBS and 3 µg/ml puromycin.

### siRNA knockdowns

siRNA(ON-TARGETplus SMARTpool) oligos towards IRS4 and IRS1 and Scrambled (control) were from Thermo Scientific. Cells at 40–50% confluency were transfected using Dharmafect 1 transfection reagent following manufacturer's instructions. Briefly, for transfection of cells in one well of a 6-well plate, siRNA oligos (100 nM final concentration) and Dharmafect 1 (10 µl) were incubated separately in 200 µl of Opti-MEM for 5 min, mixed gently and incubated at room temperature for a further 20 min. The mixture was added slowly to the cells and the culture media replaced with fresh after 12 h of siRNA transfection.

### Reproducibility

Results shown are representative of at least three similar experiments with the exception of Figure 1B and Figure 4E which were repeated twice.

## Supporting Information

Figure S1 **Cells with high levels of IRS4 became dependent on it for signalling to Akt.** Cells expressing low (#1) or high #3) levels of IRS4 were transfected with control Scrambled, IRS1 (A) panel or IRS4 (B) siRNA oligos and phosphorylation of Akt was assessed under serum conditions.
(TIF)

Figure S2 **Tyrosine phosphorylation of IRS4 and IRS1.** HEK293T cells were serum starved and then treated with IGF1 (50 ng/ml, 20 min) and serum (10%, 30 min) as indicated. Phosphorylation of Akt and tyrosine phosphorylated proteins were analysed on the right hand side panel. Tyrosine phosphorylation of IRS4 and IRS1 was analysed after immunoprecipitating the endogenous IRS4 and IRS1 (left hand side panel).
(TIF)

Table S1 (XLSX)
