# Supplementary figures and images for: Effect of IRS4 Levels on PI 3-Kinase Signalling
Source: PLoS One. 2013 Sep 10;8(9):e73327. doi: 10.1371/journal.pone.0073327 (PMC3769281; doi:10.1371/journal.pone.0073327)

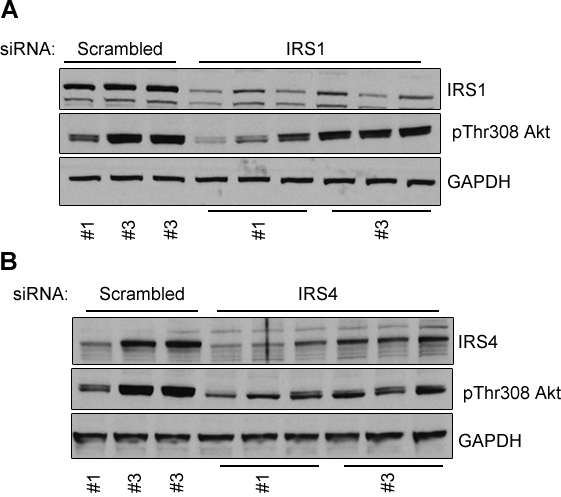

Supplement: Figure S1 — Cells with high levels of IRS4 became dependent on it for signalling to Akt. Cells expressing low (#1) or high #3) levels of IRS4 were transfected with control Scrambled, IRS1 (A) panel or IRS4 (B) siRNA oligos and phosphorylation of Akt was assessed under serum conditions. (TIF) [file pone.0073327.s001.tif]

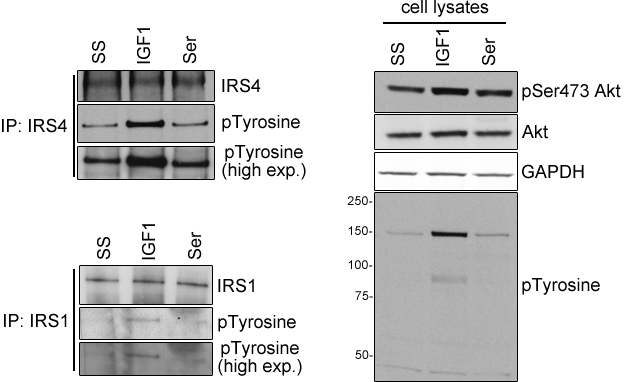

Supplement: Figure S2 — Tyrosine phosphorylation of IRS4 and IRS1. HEK293T cells were serum starved and then treated with IGF1 (50 ng/ml, 20 min) and serum (10%, 30 min) as indicated. Phosphorylation of Akt and tyrosine phosphorylated proteins were analysed on the right hand side panel. Tyrosine phosphorylation of IRS4 and IRS1 was analysed after immunoprecipitating the endogenous IRS4 and IRS1 (left hand side panel). (TIF) [file pone.0073327.s002.tif]
